# Comparison of biopsy under‐sampling and annual progression using hidden markov models to learn from prostate cancer active surveillance studies

**DOI:** 10.1002/cam4.3549

**Published:** 2020-11-06

**Authors:** Weiyu Li, Brian T. Denton, Daan Nieboer, Peter R. Carroll, Monique J. Roobol, Todd M. Morgan, Movember Foundation’s Global Action Plan Prostate Cancer Active Surveillance (GAP3) consortium

**Affiliations:** ^1^ Department of Industrial and Operations Engineering University of Michigan Ann Arbor MI USA; ^2^ Department of Urology Department of Public Health Erasmus University Medical Center Rotterdam The Netherlands; ^3^ Department of Urology UCSF ‐ Helen Diller Family Comprehensive Cancer Center San Francisco CA USA; ^4^ Department of Urology Erasmus University Medical Center Rotterdam The Netherlands; ^5^ Department of Urology University of Michigan Ann Arbor MI USA

**Keywords:** active surveillance, biopsy, biopsy under‐sampling, cancer progression, hidden Markov model, prostate cancer

## Abstract

This study aimed to estimate the rates of biopsy undersampling and progression for four prostate cancer (PCa) active surveillance (AS) cohorts within the Movember Foundation's Global Action Plan Prostate Cancer Active Surveillance (GAP3) consortium. We used a hidden Markov model (HMM) to estimate factors that define PCa dynamics for men on AS including biopsy under‐sampling and progression that are implied by longitudinal data in four large cohorts included in the GAP3 database. The HMM was subsequently used as the basis for a simulation model to evaluate the biopsy strategies previously proposed for each of these cohorts. For the four AS cohorts, the estimated annual progression rate was between 6%–13%. The estimated probability of a biopsy successfully sampling undiagnosed non‐favorable risk cancer (biopsy sensitivity) was between 71% and 80%. In the simulation study of patients diagnosed with favorable risk cancer at age 50, the mean number of biopsies performed before age 75 was between 4.11 and 12.60, depending on the biopsy strategy. The mean delay time to detection of non‐favorable risk cancer was between 0.38 and 2.17 years. Biopsy undersampling and progression varied considerably across study cohorts. There was no single best biopsy protocol that is optimal for all cohorts, because of the variation in biopsy under‐sampling error and annual progression rates across cohorts. All strategies demonstrated diminishing benefits from additional biopsies.

## Graphical abstract

There was no single best biopsy protocol in prostate cancer active surveillance that is optimal for all cohorts. The optimal biopsy strategy depends on biopsy under‐sampling error and cancer progression rate, which vary significantly across cohorts.

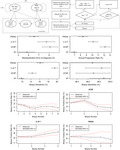

## 1 INTRODUCTION

Although early detection is key to preventing prostate cancer (PCa) death, many patients are diagnosed with low‐risk cancer that is unlikely to cause harm.^1^ Prostatectomy and radiation therapy are associated with potentially serious side effects, including incontinence, erectile dysfunction, and others.^2^ Therefore, definitive treatment of low‐risk PCa may cause more harm than good. Active surveillance (AS) is a form of expectant management, in which a switch to curative treatment can be made as a result of tumor risk reclassification at any time. AS strategies involve monitoring patients through a combination of digital rectal exams (DREs), prostate‐specific antigen (PSA) tests, selective use of imaging, and surveillance biopsies. AS defers or avoids definitive treatment until there is evidence of cancer misclassification or progression, thus reducing overtreatment of low‐risk PCa. PSA tests and DREs are minimally invasive, but they have poor predictive performance. Biopsy is the gold standard, but it involves sampling tissue from the prostate with hollow‐core needles, which can be painful, costly, and may result in infections. While PSA tests and DREs are routine elements of AS, they are far less informative than prostate biopsy for determining disease risk in this setting.

There are two main challenges when deciding the optimal biopsy plan for a given patient on AS. First, the true cancer state of each patient is not observable unless the patient is treated with radical prostatectomy, because biopsies are associated with under‐sampling error. Second, patients who start AS may later progress from favorable to non‐favorable risk over time due to cancer evolution. Moreover, the biopsy under‐sampling errors and cancer progression rates are unknown and may vary among different cohorts. A related study estimated biopsy under‐sampling error assuming no cancer progression during AS^3^ and another study that estimated progression rate assuming perfect prostate biopsy.^4^ There is one study^5^ that considered biopsy under‐sampling and PCa progression simultaneously, but it was based on a single very low‐risk cohort and did not utilize PSA or treatment outcomes for model estimation. There is no study we are aware of that considers cancer progression and biopsy misclassification across multiple cohorts.

In this study, we estimated and compared the misclassification error of favorable risk cancer at diagnosis, subsequent cancer progression rate, biopsy sensitivity and specificity, and PSA distribution in four of the most well‐known AS cohorts using the dataset (version 3.1) created by the Movember Foundation Global Action Plan Prostate Cancer Active Surveillance (GAP3).^6^ We used a hidden Markov model (HMM) to estimate the stochastic model that best describes the longitudinal observational data for each of the four cohorts. We further used the estimated models as the basis for a simulation model to compare previously published biopsy protocols across the four cohorts. We analyzed the differences in model estimates across the four cohorts and validated the results using bootstrapping. Finally, we compared the mean number of biopsies for patients on AS and the mean delay time to detection of non‐favorable risk PCa for the biopsy protocols previously proposed for each of these cohorts to assess variation in outcomes across cohorts.

## 2 MATERIALS AND METHODS

### 2.1 Data

In 2014, the Movember Foundation launched the GAP3 plan initiative to create a global database tracking the selection and monitoring of men with low‐risk PCa on AS.^6^ The database records the clinical and demographic characteristics of 20,652 patients on AS from 27 established cohorts worldwide (v3.1). In this study, we chose four cohorts including the two largest AS study cohorts in the USA: Johns Hopkins (JH) hospital^7^ and University of California San Francisco (UCSF) medical center,^8^ the largest AS study in Canada: University of Toronto (U of T) medical center,^9^ and the largest AS study outside North America: the Prostate Cancer Research International Active Surveillance (PRIAS) project.^10^ These four cohorts not only include the greatest number of patients, but also have the most AS follow‐up records over time. Importantly, these cohorts have different inclusion criteria and recommended surveillance strategies. Table 1 illustrates inclusion criteria and biopsy protocols in the four cohorts. The research was approved by the Institutional Review Board at the University of Michigan.

**TABLE 1:** Inclusion criteria and biopsy protocols for four major active surveillance cohorts

Cohort	Number of Patients	Inclusion Criteria for AS	Biopsy Protocol
JH	1,434	clinical stage ≤T1c, PSA density ≤0.15, Gleason score ≤6, total positive core ≤2, single core positivity ≤50%	Biopsy every year
UCSF	1,644	clinical stage T1‐T2, PSA ≤10, Gleason score ≤6, total positive core ≤1/3 of total cores, single core positivity ≤50%	Biopsy 1 year after diagnosis, then every 1 to 2 years
U of T	1,243	clinical stage T1c/T2a, PSA ≤10, Gleason score ≤6	Biopsy 1 year after diagnosis, then every 3 years
PRIAS	4,700	clinical stage T1c/T2, PSA ≤10, PSA density ≤0.2, Gleason score ≤6, total positive core ≤2	Biopsy 1 year after diagnosis, then every 3 years

### 2.2 Natural history models based on HMMs

We formulated an HMM to determine the misclassification error of favorable risk cancer at diagnosis due to diagnosis test error, annual progression rate to non‐favorable risk cancer, and follow‐up biopsy under‐sampling error for patients on AS in each of the four studies. HMMs are well suited to this analysis because PCa progresses stochastically over time, and the true cancer state cannot be observed directly (it is hidden due to the imperfect accuracy of PSA testing and prostate biopsies) unless the patient is treated by radical prostatectomy. We defined the *favorable risk cancer state* as the cancer state that meets the inclusion criteria in each cohort (Table 1) and defined the *non*‐*favorable risk cancer state* as any cancer state that does not meet the criteria, and thus represent cancer states for which patients may consider treatment rather than AS. Table 1 shows that the definitions of favorable and non‐favorable risk cancer vary by cohort.

**FIGURE 1:**
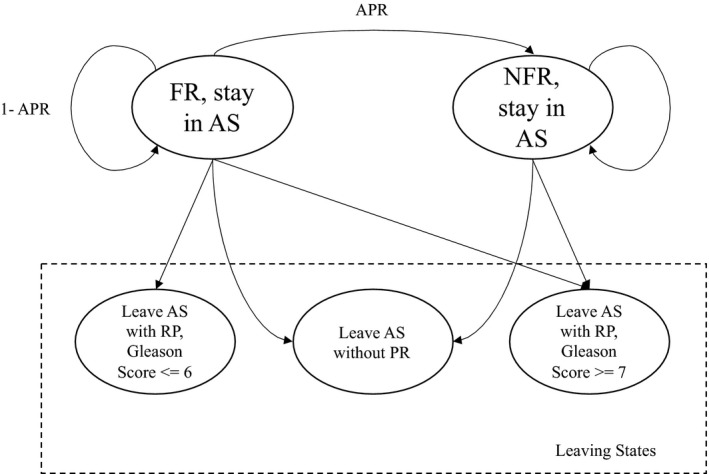
State transition diagram of PCa in the context of AS. There are two hidden states and three observable (leaving) states in the formulated HMM. Abbreviations: APR, annual progression rate; FR, favorable risk; NFR, non‐favorable risk; RP, radical prostatectomy

By definition of these cohorts, all patients were diagnosed with favorable risk PCa and initiated AS as their initial management. However, due to the potential measurement error in DREs, PSA test, and biopsy, some patients starting AS were actually in the non‐favorable risk cancer state at the time of diagnosis. We use the term *misclassification at diagnosis* to refer to instances where a patient with non‐favorable risk PCa is incorrectly diagnosed with favorable risk PCa at the time of initiating AS. The probability of misclassification at diagnosis was estimated by the initial distribution of the HMM. Every year after initiating AS, patients may also progress from favorable risk cancer to non‐favorable risk cancer with some annual progression rate, which determines the transition probability matrix in the proposed HMM. Figure 1 shows the state transition diagram of PCa in the context of AS.

The observations used to fit the HMM were PSA level and biopsy. We did not consider other covariates including clinical stage, total positive cores in biopsy, single‐core positivity in biopsy, and MRI scan because of the lack of data. In all four studies, PSA tests were routinely performed at office visits (every 3–6 months), while biopsies were performed at most once per year and often less frequently because of the design of biopsy protocols or other patient and clinical factors. Therefore, in our model, we set the frequency of test outcomes to be annual, which means we only used the most recent PSA test and one biopsy result at the end of each calendar year as observations for this annual time period. We also defined a null observation for instances of a missing test result. Given that biopsies are not perfect, we use the term *biopsy under*‐*sampling* to denote the circumstance where there was a Gleason score 6 or lower biopsy result in a patient with (hidden) non‐favorable risk cancer. The biopsy *sensitivity* (defined as rate of biopsy Gleason score 7 or higher while in the non‐favorable risk cancer state) and *specificity* (defined as rate of biopsy Gleason score 6 or lower while in favorable risk cancer state), and the distribution of the PSA testing result were estimated by the observation probability distributions in the HMM. Finally, every year, the patient might leave AS with or without treatment. If the patient left AS and underwent the radical prostatectomy, then his true cancer grade (Gleason score) was available based on post‐radical prostatectomy pathology. Otherwise, the patient was assumed to leave AS without knowledge of the true cancer grade. Given this context, we defined the leaving states of the HMM as follows: (a) *leaving AS with true Gleason score 6 or lower based on prostatectomy pathology*, (b) *leaving AS with true Gleason score 7 or higher based on prostatectomy pathology*, and (c) *leaving AS without radical prostatectomy*. The probabilities of entering the leaving states were also elements of the transition probability matrix.

We used the Baum‐Welch algorithm to fit the proposed HMM.^11^ The Baum‐Welch algorithm is a special form of the standard EM (expectation‐maximization) algorithm,^12^ which iteratively updates the estimates of model parameters that locally maximize the likelihood function of given sequences of observations. To avoid local maxima, we randomly chose different starting points of the parameters before running the iterations, and then picked the set of estimated parameters with the largest likelihood function as the final estimates. For different cohorts, we fitted different HMMs with the same model structure but different parameters.

### 2.3 Statistical analysis and validation

To estimate confidence intervals (CIs) of the estimated model parameters in different cohorts, we used the non‐parametric bootstrap method to compute the standard errors of estimated parameters.^13^ Specifically, for each cohort, we first randomly sampled patients with replacement. The number of sampled patients was equal to the sample size of the cohort. For each bootstrap sample, we then fitted an HMM using the observation sequences of the bootstrap sample. We drew 100 bootstrap samples and used the empirical standard errors and confidence intervals as the estimates of the standard errors and confidence intervals of the estimated parameters in this cohort. We repeated the same steps for all four cohorts.

We focused on internal validation in this study, because different cohorts had different study inclusion criteria. We validated the estimated models by comparing the observed and estimated distributions of the results of PSA test and biopsy. For PSA results, we compared the empirical and estimated distribution for both favorable risk cancer and non‐favorable risk cancer patients. For biopsy results, we first simulated patients’ underlying cancer states and biopsy observations (if the biopsy protocol suggested a biopsy) in each cohort using a simulation model (described in next) with the estimated model parameters. Then, we compared the observed and simulated biopsy positive rates at each biopsy time.

### 2.4 Biopsy protocols comparison by simulation model

**FIGURE 2:**
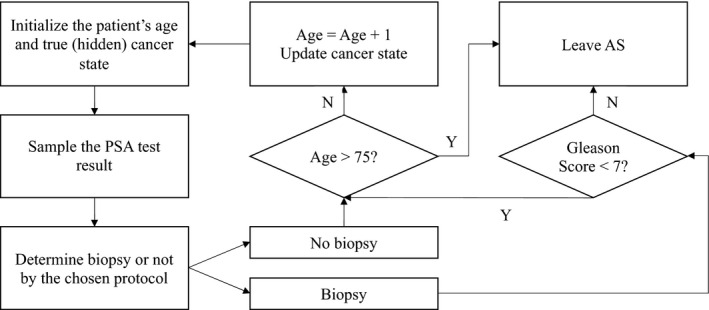
Simulation process flow for the proposed simulation model. The model parameters were determined by the estimates of the HMMs. Patients would leave the AS if they had a Gleason score 7 or higher biopsy, or they reached age 75

We used the estimated HMMs to create a simulation model for each cohort to compare the mean number of biopsies performed while on AS and the mean delay in time to detection of non‐favorable risk cancer by biopsy. Hypothetical patients in the simulation model were assumed to be diagnosed with favorable risk cancer at age 50 in different cohorts when using the different biopsy protocols described in Table 1. For each patient, we first sampled his initial cancer state when diagnosed with favorable risk cancer at age 50 according to the misclassification error at diagnosis as estimated by the HMM, and initiating AS. Second, for the next annual time‐point, we simulated his new cancer state based on the previous cancer state and the estimated annual cancer progression rate. With the simulated cancer state, we then sampled the patient's PSA result using the estimated PSA probability density distribution. If a biopsy was indicated according to the chosen protocol, we sampled the biopsy result based on the estimated sensitivity and specificity of the biopsy obtained from the HMM for the cohort. If the sampled biopsy Gleason score was greater or equal to 7 at that time point, then the patient left AS; otherwise, the patient continued on AS for another year. Patients reaching age 75 were assumed to stop AS and transit to watchful waiting. The details of the simulation process flow are shown in Figure 2.

With the simulated true cancer states and biopsy results for all patients at all time periods, the mean number of biopsies performed while on AS was calculated as the average number of follow‐up biopsies performed from initiating AS (age 50) to leaving AS (age 75 or a Gleason score 7 or higher biopsy), while the mean delay in time to detection of non‐favorable risk cancer was calculated as the average difference between the time of the first sampled non‐favorable risk cancer state and the time of a sampled Gleason score 7 or higher biopsy results for all patients. The number of sampled patients was set to 10,000 for each cohort and each protocol.

## 3 RESULTS

### 3.1 Data

Table 2 summarizes patient characteristics at the time of diagnosis for patients with at least one follow‐up year on AS. The means of age at diagnosis were similar in all four cohorts except UCSF, where patients were younger than compared to the other three cohorts. In terms of PSA levels and biopsy results, JH enrolled patients with lower PSA, lower maximum percentage of cancer in biopsy cores, and lower Gleason score than other three cohorts. UCSF and University of Toronto medical centers enrolled patients with the highest PSA level and percentage of patients with Gleason 3 + 4 = 7 or greater cancer. Additional information about patient characteristics at the time of each biopsy in AS can be found in Table S1–S4 in Appendix B.

**TABLE 2:** Patient characteristics at the time of diagnosis

Cohort	JH	UCSF	U of T	PRIAS
Patients, n	1434	1644	1243	4700
Age at biopsy, year, mean (SD)	66 (6.1)	63 (7.6)	66 (8.1)	66 (6.9)
Months since diagnosis, month, mean (SD)	0 (0)	0 (0)	0 (0)	0 (0)
PSA, ng/mL, mean (SD)	5.2 (2.9)	6.4 (4.1)	6.2 (3.1)	5.9 (2.1)
No. of biopsy cores used, median (range)	12 (6‐58)	14 (1‐50)	10 (1‐190)	12 (3‐25)
Maximum % of cancer in any one core (SD)	10 (14.8)	26 (20.8)	21 (20)	NA (NA)
% of cores with cancer	12 (7.1)	17 (13.8)	23 (18.1)	13 (6.7)
ISUP grade group, # (%)				
No cancer	0 (0)	0 (0)	0 (0)	0 (0)
1 (3 + 3)	1428 (99.6)	1437 (87.4)	1104 (88.8)	4657 (99.1)
2 (3 + 4)	6 (0.4)	178 (10.8)	139 (11.2)	42 (0.9)
3 (4 + 3)	0 (0)	25 (1.5)	0 (0)	1 (0)
4 (4 + 4)	0 (0)	4 (0.2)	0 (0)	0 (0)
5 (9, 10)	0 (0)	0 (0)	0 (0)	0 (0)
NA	0 (0)	0 (0)	9 (0.7)	3 (0.1)
Medium/High‐grade cancer (%)	6 (0.4)	207 (12.6)	139 (11.2)	43 (0.9)

Abbreviations: ISUP, International Society of Urologic Pathologists; NA, not available; SD, standard deviation.

As we can see from Table 2, some patients with medium/high‐grade (non‐favorable risk) cancer were also included in the AS. Those patients were generally older patients who continued on AS instead of moving on to treatment. For the purpose of our study, we removed those patients with medium/high‐grade cancer at diagnosis when fitting the HMMs.

### 3.2 HMM analysis and validation

**FIGURE 3:**
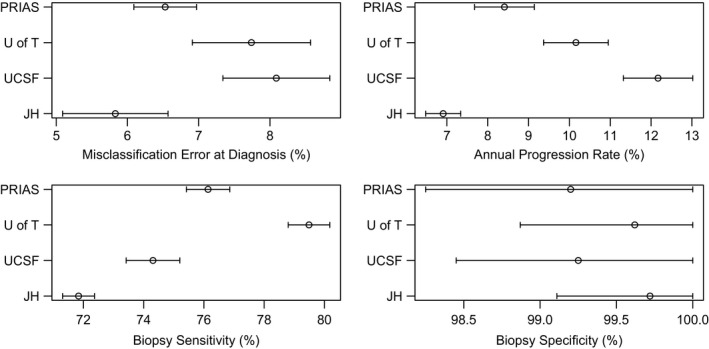
Estimated standard errors and 95% confidence intervals for the parameters in HMMs by the bootstrap method. All misclassification errors at diagnosis, annual cancer progression rates, and (1‐ biopsy sensitivity)’s are statistically significantly greater than 0

Table 3 and Figure 3 show the estimates of the most important HMM parameters for each cohort and the 95% confidence intervals estimated via the bootstrap method. The differences in the estimated annual cancer progression rates and biopsy sensitivities for distinct cohorts were statistically significant (*p* < 0.05). The estimated annual progression rate from favorable risk cancer to non‐favorable risk cancer was highest in UCSF and lowest in JH. Biopsy sensitivity was highest in PRIAS, with the highest proportion of non‐favorable risk cancer patients correctly identified on biopsy; while JH had a slightly lower biopsy sensitivity than other three cohorts. In terms of misclassification errors at diagnosis, the proportion of patients considered to have non‐favorable risk cancer at diagnosis was highest in UCSF and lowest in JH. All estimated biopsy specificities were close to 100%. In addition, based on the estimated 95% confidence intervals by bootstrapping, the estimated miss‐classification errors at diagnosis, annual cancer progression rates, and (1‐biopsy sensitivity)’s in the four cohorts are all statistically significantly greater than zero.

**TABLE 3:** Estimated parameters by the HMMs for different cohorts

Center	Number of patients	Misclassification error at diagnosis	Annual progression rate	Biopsy sensitivity	Biopsy specificity
JH	1428	5.83%	6.91%	71.84%	99.72%
UCSF	1437	8.09%	12.17%	74.31%	99.25%
U of T	1104	7.74%	10.16%	79.49%	99.62%
PRIAS	4657	6.53%	8.41%	76.14%	99.20%

For the estimates of the PSA distributions, we assumed that the logarithm of the PSA result follows a mixture of two Gaussian distributions. The details of the estimated parameters for the mixture distribution can be found in Table S5 and S6 in Appendix B.

**FIGURE 4:**
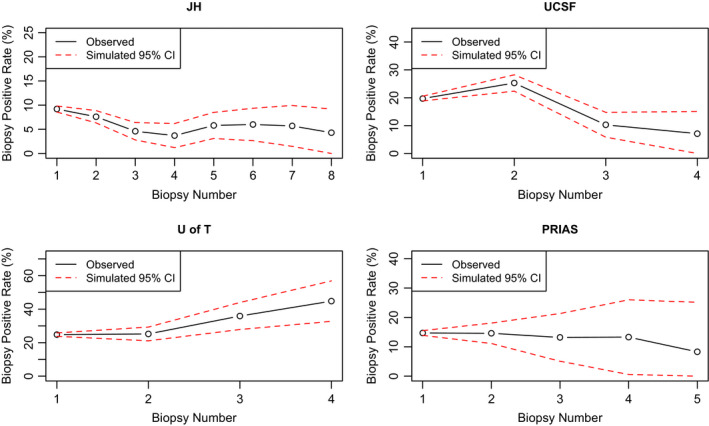
Comparison of observed and simulated biopsy positive rates at each biopsy time for different cohorts. All observed biopsy detection rates fell into the 95% CIs of the simulated detection rates

We validated our models by comparing the biopsy positive rates and PSA probability density functions between observed and simulated data. Figure 4 shows the comparisons between observed and simulated biopsy positive rates for different cohorts, which were calculated as the number of patients with a positive biopsy (Gleason score 7 or higher biopsy) results divided by the total number of patients who underwent biopsy in the observed and simulated datasets, at each biopsy time point. The observed positive biopsy rates all fell into the 95% confidence intervals of the simulated biopsy positive rates. The comparisons of PSA distributions are shown in Figure S1–S4 in Appendix B.

### 3.3 Comparison of biopsy protocols

We simulated a population of 5000 patients for each cohort and each biopsy protocol using the simulation model described in Figure 2. Each patient was assumed to be diagnosed as favorable risk cancer and enter AS at age 50. We compared the frequency of biopsy and the mean delay in time to detection of non‐favorable risk cancers between the time of diagnosis (age 50) and the end of AS. Table 4 shows the simulation results for all protocols in fours cohorts. In each cohort, the protocol employing fewer biopsies was associated with a longer late detection time on average. Also, if we compare the differences in the mean number of biopsies used and mean delay in detection by biopsy between different protocols, we can see the benefit from more frequent biopsies was diminishing.

**TABLE 4:** Comparisons of the mean number of biopsies used and average late detection time by biopsy between the time of diagnosis and the end of AS for different protocols in different cohorts by the proposed simulation model

Cohort	JH			UCSF			U of T			PRIAS		
Biopsy protocol	JH	UCSF	U of T	JH	UCSF	U of T	JH	UCSF	U of T	JH	UCSF	U of T
Mean number of biopsies	12.6	7.1	5.3	8.7	5.3	4.1	9.7	5.8	4.4	11.1	6.4	4.9
Average late detection time by biopsy (month)	4.5	13.9	22.9	5.0	15.2	26.0	3.8	12.7	21.7	4.0	13.2	22.3

## 4 DISCUSSION

We estimated the misclassification error at diagnosis, the annual cancer progression rate, the sensitivity and specificity of biopsy, and the distribution of PSA in four PCa AS cohorts part of the GAP3 consortium: JH, UCSF, U of T, and PRIAS. With the estimated HMMs, we then compared the mean number of biopsies performed versus late detection of cancer progression by biopsy when following different published biopsy protocols in four cohorts using a series of simulations. As expected, in each cohort, the biopsy protocol that recommended more frequent biopsies was associated with shorter time to reclassification. Our results show that because of the considerable variation in biopsy under‐sampling error and annual progression rates across cohorts, there was no single best biopsy protocol that is optimal for all cohorts. Moreover, in each cohort, the biopsy protocol that recommended more frequent biopsies was associated with shorter time to reclassification, while the benefit from additional biopsies was diminishing.

Other studies have also tried to quantify the most important factors associated with testing errors and cancer progression rate on AS. Coley et al.^3^ proposed a Bayesian hierarchical model that included PSA and biopsy as covariates to predict the latent cancer state in the JH AS cohort. They estimated the misclassification error at diagnosis to be between 20% and 31%, and the biopsy sensitivity to be 62%. The reason why their measurement error was much higher than ours was that they assumed there was no cancer progression during AS for any patient. For our fitted HMMs, we do see that estimates of both cancer progression rate and biopsy under‐sampling error are statistically significantly greater than 0, as the bootstrapping 95% confidence intervals do not include 0. Thus, if we apply the bootstrap‐*t* hypothesis test method discussed in Efron and Tibshirani^14^ to the estimates of both cancer progression rate and biopsy under‐sampling error, we can reject the null hypothesis that the estimated parameter is equal to 0 with the type I error less than 5%. Also, the definition of the biopsy sensitivity in our study, is defined with respect to the non‐favorable risk cancer state as defined in each of the studies as opposed to Gleason score alone, used by Coley and colleagues.

Another study by Barnett et al.,^5^ fit an HMM to estimate the cancer grade progression rate and biopsy under‐sampling errors in the JH AS cohort only. They estimated the annual progression rate from Gleason score 6 cancer to Gleason score 7 or higher cancer to be 4.0%; then sensitivity and specificity of biopsy to be 61.0% and 98.6%. There are a number differences in their approach compared to our study. For example, they did not incorporate PSA observations or observations of radical prostatectomy or alternative treatment options, which can reveal the true cancer states, for patients to leave AS. Moreover they considered only the JH cohort which was a very low risk patient cohort. Thus, we believe our model in this study was more informative than their model. A study by Inoue et al.,^4^ which compared the biopsy upgrading rates in four PCa AS cohorts including JH, UCSF, U of T, and Canary prostate cancer active surveillance study cohorts found a statistically significant difference in biopsy upgrading risk for different cohorts. However, they did not account for possible biopsy Gleason score false‐negative result and misclassification error.

In our result of the HMMs for four different cohorts, based on the bootstrapped standard errors of the estimated parameters, all the mis‐classification errors at diagnosis, annual cancer grade progression rates, and biopsy false‐negative rates were statistically significantly greater than zero. This validates our assumptions about the non‐zero progression rate in contrast to the above‐referenced study by Coley et al.^3^ that assumes no progression, and the imperfect biopsy sensitivity in contrast to the study by Inoue et al.^4^ that assumes zero misclassification error and zero biopsy false‐negative rate. All biopsy specificities were close to 100%, indicating it was very rare that a patient in favorable risk cancer state would have a biopsy Gleason sum 7 or higher. For mis‐classification errors at the time of diagnosis and annual grade progression rates, we found that the estimates in the UCSF and U of T cohorts were greater than the estimates in JH and PRIAS cohorts. This was consistent with the fact that the UCSF and U of T cohorts included higher‐risk patients than other two cohorts, which can also be seen in the summary statistics of PSA density, maximum percentage of cancer in any one core, and percentage of cores with cancer at the time of diagnosis in Table 2. For the biopsy sensitivities, we saw that JH cohort had the lowest estimate while the U of T cohort had the highest one. Our conjecture was that patients with lower risk had smaller tumors in general, so that they were harder to detect by biopsy if they were in non‐favorable risk cancer state. Other possible reasons for such differences might include the different definition of favorable and non‐favorable risk states, and the difference in the urologist practice when performing the tests in different cohorts.

Our simulation study compared three published biopsy protocols in different cohorts. Within each cohort, the protocol that recommended more biopsies had less late detection years of non‐favorable risk cancer by biopsy. However, we saw that the benefit in terms of early detection was diminishing along with the increasing number of biopsies. There was no single optimal protocol that recommended fewer biopsies but could detect non‐favorable risk cancer earlier, in any cohort. Two main reasons are: first, the model parameters estimated by the HMMs and used in the simulation model were statistically significantly different for different cohorts; second, there were two competing objectives when comparing the protocols that are minimizing the number of biopsies and minimizing the late detection time by biopsy.

There were some notable limitations in our study. First, we reduced a complex disease (PCa) to a two‐state (favorable and unfavorable risk) stochastic model with two outputs of the disease (results of PSA test and biopsy) as informative observations. However, although such models cannot capture all details about the disease, it consistently discriminates health states on the basis of the most significant factors defining study inclusion for each cohort. Second, our proposed HMM included the null observation of biopsy as non‐informative missingness. In other words, we assumed no difference between a missed biopsy by the design of the study, and a missed biopsy result for other reasons (e.g. patient preference, data lost to follow‐up). However, by using the null observation to denote the biopsy missingness in the HMM, we mitigated bias in our estimates of the model parameters. Finally, another way to monitor PCa in recent AS protocols is by magnetic resonance imaging (MRI) scans, but it was not considered in this study due to the lack of sufficient longitudinal data.

The above limitations notwithstanding, our study quantified the most important factors in four PCa AS cohorts, providing a number of insights into the role of different study designs and populations on AS. We found there was no single optimal biopsy protocol across cohorts and we provided evidence that there may be considerable variation in characteristics of PCa across cohorts. This is likely explained by some combination of factors including: 1) differences in disease dynamics between the different cohorts due to variations in the inclusion criteria, and thus different definitions of favorable vs. non‐favorable risk PCa. and 2) variation in healthcare delivery across health systems resulting from different practices in urology and pathology.

## 5 **AUTHOR CONTRIBUTIONS**:

Weiyu Li: Conceptualization, Methodology, Software, Data curation, Validation, Formal analysis, Investigation, Visualization, Project administration, Writing – original draft, and Writing – review and editing. Brian T. Denton: Conceptualization, Methodology, Resources, Visualization, Supervision, Project administration, Funding acquisition, and Writing – review and editing. Daan Nieboer: Conceptualization, Methodology, and Writing – review and editing. Peter R. Carroll: Conceptualization, and Writing – review and editing. Monique J. Roobol: Conceptualization, and Writing – review and editing. Todd M. Morgan: Conceptualization, Methodology, and Writing – review and editing. Movember Foundation's Global Action Plan Prostate Cancer Active Surveillance (GAP3) consortium: Data curation, and project administration.

## CONFLICTS OF INTEREST

There are no conflict of interest disclosures from any author.

## Supporting information

Supplementary Material

## Data Availability

The data that support the findings of this study are available from Movember Foundation's GAP3 consortium. Restrictions apply to the availability of these data, which were used under license for this study. Data are available from the authors with the permission of Movember Foundation's GAP3 consortium.
