# Supplementary material for: Comparison of biopsy under‐sampling and annual progression using hidden markov models to learn from prostate cancer active surveillance studies
Source: Cancer Med. 2020 Nov 6;9(24):9611–9. doi: 10.1002/cam4.3549 (PMC7774732; doi:10.1002/cam4.3549)
Supplement: Supplementary file 1 — Supplementary Material [file CAM4-9-9611-s001.docx]

# Supporting Information

## Appendix A Members of The Movember Foundation’s Global Action Plan Prostate Cancer Active Surveillance (GAP3) consortium

Principle Investigators: Bruce Trock (Johns Hopkins University, The James Buchanan Brady Urological Institute, Baltimore, USA), Behfar Ehdaie (Memorial Sloan Kettering Cancer Center, New York, USA), Peter Carroll (University of California San Francisco, San Francisco, USA), Christopher Filson (Emory University School of Medicine, Winship Cancer Institute, Atlanta, USA), Jeri Kim / Christopher Logothetis (MD Anderson Cancer Centre, Houston, USA), Todd Morgan (University of Michigan and Michigan Urological Surgery Improvement Collaborative (MUSIC), Michigan, USA), Laurence Klotz (University of Toronto, Sunnybrook Health Sciences Centre, Toronto, Ontario, Canada), Tom Pickles (University of British Columbia, BC Cancer Agency, Vancouver, Canada), Eric Hyndman (University of Calgary, Southern Alberta Institute of Urology, Calgary, Canada), Caroline Moore (University College London & University College London Hospital Trust, London, UK), Vincent Gnanapragasam (University of Cambridge & Cambridge University Hospitals NHS Foundation Trust, Cambridge, UK), Mieke Van Hemelrijck (King's College London, London, UK & Guy’s and St Thomas’ NHS Foundation Trust, London, UK), Prokar Dasgupta (Guy’s and St Thomas’ NHS Foundation Trust, London, UK), Chris Bangma (Erasmus Medical Center, Rotterdam, The Netherlands/ representative of Prostate cancer Research International Active Surveillance (PRIAS) consortium), Monique Roobol (Erasmus Medical Center, Rotterdam, The Netherlands/ representative of Prostate cancer Research International Active Surveillance (PRIAS) consortium), The PRIAS study group, Arnauld Villers (Lille University Hospital Center, Lille, France), Grégoire Robert (Centre Hospitalier Universitaire de Bordeaux (CHU), Bordeaux, France), Axel Semjonow (University Hospital Muenster, Muenster, Germany), Antti Rannikko (Helsinki University and Helsinki University Hospital, Helsinki, Finland), Riccardo Valdagni (Department of Oncology and Hemato-oncology, Università degli Studi di Milano, Radiation Oncology 1 and Prostate Cancer Program, Fondazione IRCCS Istituto Nazionale dei Tumori, Milan, Italy), Antoinette Perry (University College Dublin, Dublin, Ireland), Jonas Hugosson (Sahlgrenska University Hospital, Göteborg, Sweden), Jose Rubio-Briones (Instituto Valenciano de Oncología, Valencia, Spain), Anders Bjartell (Skåne University Hospital, Malmö, Sweden), Lukas Hefermehl (Kantonsspital Baden, Baden, Switzerland), Lee Lui Shiong (Singapore General Hospital, Singapore, Singapore), Mark Frydenberg (Monash Health; Monash University, Melbourne, Australia), Yoshiyuki Kakehi / Mikio Sugimoto (Kagawa University Faculty of Medicine, Kagawa, Japan), Byung Ha Chung (Gangnam Severance Hospital, Yonsei University Health System, Seoul, Republic of Korea)

Pathologist: Theo van der Kwast (Princess Margaret Cancer Centre, Toronto, Canada).

Technology Research Partners: Wim van der Linden (Royal Philips, Eindhoven, the Netherlands), Tim Hulsen (Royal Philips, Eindhoven, the Netherlands), Cees de Jonge / Peter van Hooft (Royal Philips, Eindhoven, the Netherlands).

Advisory Regional statisticians: Mike Kattan (Cleveland Clinic, Cleveland, Ohio, USA), Ji Xinge (Cleveland Clinic, Cleveland, Ohio, USA), Kenneth Muir (University of Manchester, Manchester, UK), Artitaya Lophatananon (University of Manchester, Manchester, UK), Michael Fahey (Epworth HealthCare, Melbourne, Australia), Ewout Steyerberg (Erasmus Medical Center, Rotterdam, The Netherlands), Daan Nieboer (Erasmus Medical Center, Rotterdam, The Netherlands); Liying Zhang (University of Toronto, Sunnybrook Health Sciences Centre, Toronto, Ontario, Canada)

Executive Regional statisticians:Ewout Steyerberg (Erasmus Medical Center, Rotterdam, The Netherlands), Daan Nieboer (Erasmus Medical Center, Rotterdam, The Netherlands); Kerri Beckmann (King's College London, London, UK & Guy’s and St Thomas’ NHS Foundation Trust, London, UK), Brian Denton (University of Michigan, Michigan, USA), Andrew Hayen (University of Technology Sydney, Australia), Paul Boutros (Ontario Institute of Cancer Research, Toronto, Ontario, Canada).

Clinical Research Partners’ IT Experts: Wei Guo (Johns Hopkins University, The James Buchanan Brady Urological Institute, Baltimore, USA), Nicole Benfante (Memorial Sloan Kettering Cancer Center, New York, USA), Janet Cowan (University of California San Francisco, San Francisco, USA), Dattatraya Patil (Emory University School of Medicine, Winship Cancer Institute, Atlanta, USA), Lauren Park (MD Anderson Cancer Centre, Houston, Texas, USA), e(University of Michigan and Michigan Urological Surgery Improvement Collaborative, Ann Arbor, Michigan, USA), Alexandre Mamedov (University of Toronto, Sunnybrook Health Sciences Centre, Toronto, Ontario, Canada), Vincent LaPointe (University of British Columbia, BC Cancer Agency, Vancouver, Canada), Trafford Crump (University of Calgary, Southern Alberta Institute of Urology, Calgary, Canada), Vasilis Stavrinides (University College London & University College London Hospital Trust, London, UK), Jenna Kimberly-Duffell (University of Cambridge & Cambridge University Hospitals NHS Foundation Trust, Cambridge, UK), Aida Santaolalla (King's College London, London, UK & Guy’s and St Thomas’ NHS Foundation Trust, London, UK), Daan Nieboer (Erasmus Medical Center, Rotterdam, The Netherlands), Jonathan Olivier (Lille University Hospital Center, Lille, & Centre Hospitalier Universitaire de Bordeaux (CHU), Bordeaux, France), Tiziana Rancati (Fondazione IRCCS Istituto Nazionale dei Tumori di Milano, Milan, Italy), Helén Ahlgren (Sahlgrenska University Hospital, Göteborg, Sweden), Juanma Mascarós (Instituto Valenciano de Oncología, Valencia, Spain), Annica Löfgren (Skåne University Hospital, Malmö, Sweden), Kurt Lehmann (Kantonsspital Baden, Baden, Switzerland), Catherine Han Lin (Monash University and Epworth HealthCare, Melbourne, Australia), Hiromi Hirama (Kagawa University, Kagawa, Japan), Kwang Suk Lee (Yonsei University College of Medicine, Gangnam Severance Hospital, Seoul, Korea).

Research Advisory Committee: Guido Jenster (Erasmus MC, Rotterdam, the Netherlands), Anssi Auvinen (University of Tampere, Tampere, Finland), Anders Bjartell (Skåne University Hospital, Malmö, Sweden), Masoom Haider (University of Toronto, Toronto, Canada), Kees van Bochove (The Hyve B.V. Utrecht, Utrecht, the Netherlands), Ballentine Carter (Johns Hopkins University, Baltimore, USA – until 2018).

Management team: Sam Gledhill / Mark Buzza (Movember Foundation, Melbourne, Australia), Michelle Kouspou (Movember Foundation, Melbourne, Australia), Chris Bangma (Erasmus Medical Center, Rotterdam, The Netherlands), Monique Roobol (Erasmus Medical Center, Rotterdam, The Netherlands), Sophie Bruinsma / Jozien Helleman (Erasmus Medical Center, Rotterdam, The Netherlands).

## Appendix B Supporting Tables and Figures

Table S1: Biopsy Characteristics for patients in Johns Hopkins hospital

|  | **Biopsy** | | | | | | | | |
| --- | --- | --- | --- | --- | --- | --- | --- | --- | --- |
| **Characteristics** | Diagnosis | First | Second | Third | Fourth | Fifth | Sixth | Seventh | Eighth |
| **Patients, n** | 1434 | 1229 | 776 | 524 | 349 | 224 | 134 | 88 | 47 |
| **Age at biopsy, year, mean (SD)** | 66 (6.1) | 67 (6.2) | 67 (6.1) | 68 (5.5) | 68 (5.4) | 69 (5.3) | 70 (4.7) | 70 (4.2) | 71 (4.2) |
| **Months since diagnosis, month, mean (SD)** | 0 (0) | 14 (13.2) | 29 (16.3) | 41 (15.4) | 54 (14.4) | 68 (15) | 82 (15.5) | 96 (16.1) | 107 (14.8) |
| **PSA, ng/mL, mean (SD)** | 5.2 (2.9) | 5.3 (3.4) | 5.4 (4.4) | 5.4 (3.9) | 5.3 (3.6) | 5.6 (4.6) | 5.7 (4.5) | 5.3 (4.4) | 4.7 (3.3) |
| **No. of biopsy cores used, median (range)** | 12 (6-58) | 12 (4-31) | 12 (6-60) | 12 (6-28) | 12 (8-16) | 14 (9-24) | 14 (6-15) | 14 (6-15) | 14 (8-14) |
| **Maximum % of cancer in any one core (SD)** | 10 (14.8) | 22 (24.6) | 17 (20.8) | 13 (18.7) | 18 (21.1) | 17 (18.1) | 14 (17.9) | 18 (19.4) | 15 (17.9) |
| **% of cores with cancer** | 12 (7.1) | 10 (12.4) | 7 (10.3) | 6 (8.9) | 6 (8.9) | 6 (8) | 6 (8.7) | 7 (8.7) | 7 (8.9) |
| **Gleason group, # (%)** |  |  |  |  |  |  |  |  |  |
| **No cancer** | 0 (0) | 519 (42.2) | 386 (49.7) | 289 (55.2) | 191 (54.7) | 127 (56.7) | 72 (53.7) | 43 (48.9) | 23 (48.9) |
| **1 (3 + 3)** | 1428 (99.6) | 594 (48.3) | 330 (42.5) | 209 (39.9) | 145 (41.5) | 84 (37.5) | 54 (40.3) | 40 (45.5) | 22 (46.8) |
| **2 (3 + 4)** | 6 (0.4) | 76 (6.2) | 42 (5.4) | 12 (2.3) | 9 (2.6) | 8 (3.6) | 7 (5.2) | 2 (2.3) | 2 (4.3) |
| **3 (4 + 3)** | 0 (0) | 23 (1.9) | 11 (1.4) | 11 (2.1) | 3 (0.9) | 4 (1.8) | 1 (0.7) | 2 (2.3) | 0 (0) |
| **4 (4 + 4)** | 0 (0) | 11 (0.9) | 4 (0.5) | 0 (0) | 1 (0.3) | 1 (0.4) | 0 (0) | 1 (1.1) | 0 (0) |
| **5 (9, 10)** | 0 (0) | 3 (0.2) | 2 (0.3) | 1 (0.2) | 0 (0) | 0 (0) | 0 (0) | 0 (0) | 0 (0) |
| **NA** | 0 (0) | 3 (0.2) | 1 (0.1) | 2 (0.4) | 0 (0) | 0 (0) | 0 (0) | 0 (0) | 0 (0) |
| **Medium/High-grade cancer (%)** | 6 (0.4) | 113 (9.2) | 59 (7.6) | 24 (4.6) | 13 (3.7) | 13 (5.8) | 8 (6) | 5 (5.7) | 2 (4.3) |

Table S2: Biopsy Characteristics for patients in UCSF medical center

|  | **Biopsy** | | | | | |
| --- | --- | --- | --- | --- | --- | --- |
| **Characteristics** | Diagnosis | First | Second | Third | Fourth | Fifth |
| **Patients, n** | 1644 | 279 | 99 | 39 | 14 | 4 |
| **Age at biopsy, year, mean (SD)** | 63 (7.6) | 64 (7.7) | 65 (7.2) | 67 (7.2) | 69 (6.8) | 69 (3.8) |
| **Months since diagnosis, month, mean (SD)** | 0 (0) | 25 (24.8) | 42 (26.5) | 63 (32.1) | 82 (22.2) | 109 (14.4) |
| **PSA, ng/mL, mean (SD)** | 6.4 (4.1) | 5.8 (5.8) | 5.6 (3.5) | 8.7 (18) | 6 (4.8) | 4.1 (2.5) |
| **No. of biopsy cores used, median (range)** | 14 (1-50) | 16 (2-31) | 17 (4-25) | 16 (5-27) | 17 (14-26) | 17.5 (14-22) |
| **Maximum % of cancer in any one core (SD)** | 26 (20.8) | 5 (7.3) | 5 (6.9) | 4 (4.9) | 5 (5.1) | 5 (5) |
| **% of cores with cancer** | 17 (13.8) | 17 (16.7) | 17 (16.8) | 14 (14.8) | 15 (14.2) | 16 (11.8) |
| **Gleason group, # (%)** |  |  |  |  |  |  |
| **No cancer** | 0 (0) | 72 (25.8) | 27 (27.3) | 11 (28.2) | 3 (21.4) | 0 (0) |
| **1 (3 + 3)** | 1437 (87.4) | 152 (54.5) | 47 (47.5) | 24 (61.5) | 10 (71.4) | 3 (75) |
| **2 (3 + 4)** | 178 (10.8) | 39 (14) | 20 (20.2) | 2 (5.1) | 1 (7.1) | 1 (25) |
| **3 (4 + 3)** | 25 (1.5) | 12 (4.3) | 3 (3) | 2 (5.1) | 0 (0) | 0 (0) |
| **4 (4 + 4)** | 4 (0.2) | 3 (1.1) | 1 (1) | 0 (0) | 0 (0) | 0 (0) |
| **5 (9, 10)** | 0 (0) | 1 (0.4) | 1 (1) | 0 (0) | 0 (0) | 0 (0) |
| **NA** | 0 (0) | 0 (0) | 0 (0) | 0 (0) | 0 (0) | 0 (0) |
| **Medium/High-grade cancer (%)** | 207 (12.6) | 55 (19.7) | 25 (25.3) | 4 (10.3) | 1 (7.1) | 1 (25) |

Table S3: Biopsy Characteristics for patients in Toronto medical center

|  | **Biopsy** | | | | | |
| --- | --- | --- | --- | --- | --- | --- |
| **Characteristics** | Diagnosis | First | Second | Third | Fourth | Fifth |
| **Patients, n** | 1243 | 911 | 385 | 131 | 29 | 4 |
| **Age at biopsy, year, mean (SD)** | 66 (8.1) | 67 (8.2) | 68 (7.6) | 69 (7.4) | 70 (7.3) | 69 (10.4) |
| **Months since diagnosis, month, mean (SD)** | 0 (0) | 22 (17.5) | 58 (25) | 97 (31.7) | 136 (39.2) | 148 (47.2) |
| **PSA, ng/mL, mean (SD)** | 6.2 (3.1) | 7.7 (6.3) | 10 (13.1) | 9.3 (8.2) | 11.7 (9.3) | NaN (NA) |
| **No. of biopsy cores used, median (range)** | 10 (1-190) | 10 (2-27) | 10 (3-250) | 10 (5-170) | 10 (5-13) | 7 (6-14) |
| **Maximum % of cancer in any one core (SD)** | 21 (20) | 32 (26) | 33 (25.6) | 39 (27.1) | 44 (23.9) | 60 (NA) |
| **% of cores with cancer** | 23 (18.1) | 24 (24.5) | 25 (28.9) | 33 (31.2) | 38 (38.5) | 7 (14.3) |
| **Gleason group, # (%)** |  |  |  |  |  |  |
| **No cancer** | 0 (0) | 226 (24.8) | 128 (33.2) | 29 (22.1) | 8 (27.6) | 3 (75) |
| **1 (3 + 3)** | 1104 (88.8) | 400 (43.9) | 151 (39.2) | 52 (39.7) | 7 (24.1) | 0 (0) |
| **2 (3 + 4)** | 139 (11.2) | 160 (17.6) | 64 (16.6) | 33 (25.2) | 9 (31) | 1 (25) |
| **3 (4 + 3)** | 0 (0) | 48 (5.3) | 24 (6.2) | 11 (8.4) | 2 (6.9) | 0 (0) |
| **4 (4 + 4)** | 0 (0) | 9 (1) | 5 (1.3) | 0 (0) | 1 (3.4) | 0 (0) |
| **5 (9, 10)** | 0 (0) | 9 (1) | 4 (1) | 3 (2.3) | 1 (3.4) | 0 (0) |
| **NA** | 9 (0.7) | 72 (7.9) | 9 (2.3) | 3 (2.3) | 1 (3.4) | 0 (0) |
| **Medium/High-grade cancer (%)** | 139 (11.2) | 226 (24.8) | 97 (25.2) | 47 (35.9) | 13 (44.8) | 1 (25) |

Table S4: Biopsy Characteristics for patients in the PRIAS project

|  | **Biopsy** | | | | | | |
| --- | --- | --- | --- | --- | --- | --- | --- |
| **Characteristics** | Diagnosis | First | Second | Third | Fourth | Fifth | Sixth |
| **Patients, n** | 4700 | 3535 | 1226 | 342 | 90 | 12 | 3 |
| **Age at biopsy, year, mean (SD)** | 66 (6.9) | 67 (6.9) | 68 (6.9) | 69 (6.7) | 70 (6.5) | 72 (7.2) | 70 (7.9) |
| **Months since diagnosis, month, mean (SD)** | 0 (0) | 14 (8) | 41 (14.3) | 63 (20.1) | 77 (22.2) | 84 (19.8) | 87 (18.3) |
| **PSA, ng/mL, mean (SD)** | 5.9 (2.1) | 6.1 (3.3) | 6.8 (3.7) | 7.3 (4.2) | 8.1 (4.3) | 8.8 (3.4) | 13.2 (1.6) |
| **No. of biopsy cores used, median (range)** | 12 (3-25) | 12 (3-25) | 12 (2-25) | 12 (3-25) | 12 (6-25) | 10 (8-12) | 12 (10-12) |
| **Maximum % of cancer in any one core (SD)** | NA (NA) | NA (NA) | NA (NA) | NA (NA) | NA (NA) | NA (NA) | NA (NA) |
| **% of cores with cancer** | 13 (6.7) | 12 (14.8) | 11 (13.5) | 11 (15.6) | 9 (10.9) | 7 (11) | 40 (19.2) |
| **Gleason group, # (%)** | NA | NA | NA | NA | NA | NA | NA |
| **No cancer** | 0 (0) | 1319 (37.3) | 493 (40.2) | 158 (46.2) | 35 (38.9) | 8 (66.7) | 0 (0) |
| **1 (3 + 3)** | 4657 (99.1) | 1668 (47.2) | 540 (44) | 136 (39.8) | 42 (46.7) | 3 (25) | 1 (33.3) |
| **2 (3 + 4)** | 42 (0.9) | 374 (10.6) | 112 (9.1) | 33 (9.6) | 7 (7.8) | 0 (0) | 2 (66.7) |
| **3 (4 + 3)** | 1 (0) | 90 (2.5) | 31 (2.5) | 7 (2) | 4 (4.4) | 1 (8.3) | 0 (0) |
| **4 (4 + 4)** | 0 (0) | 46 (1.3) | 30 (2.4) | 4 (1.2) | 1 (1.1) | 0 (0) | 0 (0) |
| **5 (9, 10)** | 0 (0) | 9 (0.3) | 6 (0.5) | 1 (0.3) | 0 (0) | 0 (0) | 0 (0) |
| **NA** | 3 (0.1) | 30 (0.8) | 14 (1.1) | 3 (0.9) | 1 (1.1) | 0 (0) | 0 (0) |
| **Medium/High-grade cancer (%)** | 43 (0.9) | 519 (14.7) | 179 (14.6) | 45 (13.2) | 12 (13.3) | 1 (8.3) | 2 (66.7) |

Table S5: Estimated of the mixture Gaussian distribution of the log(PSA) in different medical centers.

| Center | Prob. Of C1 in FR state | Prob. Of C1 in NFR state | Mean of C1 | Mean of C2 | SD of C1 | SD of C2 |
| --- | --- | --- | --- | --- | --- | --- |
| Johns Hopkins | 0.4730 | 0.3381 | 1.09 | 2.04 | 0.95 | 0.55 |
| UCSF | 0.0602 | 0.0434 | 1.15 | 2.15 | 1.20 | 0.45 |
| Toronto | 1.0000 | 0.2650 | 1.49 | 2.37 | 0.97 | 1.74 |
| PRIAS | 0.2238 | 0.1620 | 1.45 | 2.16 | 0.90 | 0.44 |

Table S6: Estimated PSA distribution in different cohorts.

| Range of PSA (ng/mL) | | < 4 | [4, 10] | >10 |
| --- | --- | --- | --- | --- |
| Johns Hopkins | FR Cancer | 0.3552 | 0.4311 | 0.2137 |
|  | NFR Cancer | 0.2868 | 0.4706 | 0.2426 |
| UCSF | FR Cancer | 0.0768 | 0.5680 | 0.3552 |
|  | NFR Cancer | 0.0678 | 0.5736 | 0.3586 |
| Toronto | FR Cancer | 0.4573 | 0.3422 | 0.2005 |
|  | NFR Cancer | 0.3312 | 0.2368 | 0.4320 |
| PRIAS | FR Cancer | 0.1361 | 0.5357 | 0.3282 |
|  | NFR Cancer | 0.1094 | 0.5501 | 0.3405 |

Abbreviations: FR, favorable risk; NFR, non-favorable risk.

Figure S1: Observed and estimated density plots of the PSA in Johns Hopkins hospital

Figure S2: Observed and estimated density plots of the PSA in UCSF medical center

Figure S3: Observed and estimated density plots of the PSA in University of Toronto medical center

Figure S4: Observed and estimated density plots of the PSA in PRIAS dataset
